# Quantifying cytoskeletal protein interactions with far Western blotting

**DOI:** 10.3389/fmolb.2026.1761723

**Published:** 2026-02-12

**Authors:** Marcelo M. Melo, Elizabeth S. Phillippi, Mark L. Schultz, Susan Q. Shen, Hatem El-Shanti

**Affiliations:** 1 Department of Psychiatry, Carver College of Medicine, University of Iowa, Iowa City, IA, United States; 2 Department of Pediatrics, Carver College of Medicine, University of Iowa, Iowa City, IA, United States

**Keywords:** binding affinity, cytoskeletal proteins, far Western blot, keratins, protein–protein interaction

## Abstract

The study of cytoskeletal proteins has been limited by some of their intrinsic properties, such as fixed intracellular localization and insolubility, which limit the implementation of many protein–protein interaction assays. Far Western blotting is a powerful biochemical technique used to detect and quantify protein–protein interactions on a membrane, addressing limitations in cytoskeletal research. This method combines traditional Western blotting with protein overlay assays to identify and characterize binding partners in complex samples. Here, we present a detailed protocol for the use of far Western blotting to probe keratin–keratin interactions, including instructions for protein sample preparation, electrophoretic separation, membrane transfer, protein labeling, buffer dilutions, and detection steps. We further extend the use of far Western blotting to compare relative binding and enhance assay sensitivity by incorporating a Western blot as a loading control. This approach enables the analysis of samples that are too dilute for reliable membrane staining. Key considerations for enhancing specificity and sensitivity are discussed, along with troubleshooting tips. This approach offers a versatile tool for studying protein interaction networks, providing valuable insights into cellular signaling and molecular mechanisms, and can be conducted in any laboratory capable of performing a Western blot.

## Introduction

The study of protein–protein interactions (PPIs) is crucial for understanding how proteins function within a cell. PPIs underlie a wide range of biological processes, from cellular organization and immunological response to metabolic and developmental control, and have become a cornerstone of system biology (Rao et al., 2014; Akbarzadeh et al., 2024). Today, several approaches for detecting PPIs are routinely performed both biochemically and in cellular models. For living cells, some of the commonly implemented approaches include the yeast two-hybrid (Y2H) system, fluorescence-resonance energy transfer (FRET), bioluminescence resonance energy transfer (BRET), the synthetic lethality method, and APEX proximity labeling (Wu et al., 2007; Lobingier et al., 2017). There are also numerous biochemical approaches, such as tandem affinity purification, affinity chromatography, surface plasmon resonance (SPR), protein microarrays, dot blots, co-immunoprecipitation (co-IP), pull-down assays, protein fragment complementation, phage display, X-ray crystallography, and NMR spectroscopy (Wu et al., 2007; Rao et al., 2014).

The advent of these approaches has facilitated the study of PPIs for a wide range of proteins. However, the study of PPIs in cytoskeletal proteins remains a major challenge. Cytoskeletal proteins are present in all three domains of life and involved in various functions, including cell shape, division, movement, apoptosis, differentiation, and signaling (Ramaekers and Bosman, 2004; Wickstead and Gull, 2011). In humans, many diseases have been associated with abnormalities in cytoskeletal proteins, including cardiovascular disease, neurodegeneration, cancer, liver cirrhosis, pulmonary fibrosis, and skin disorders (Ramaekers and Bosman, 2004). The application of PPI tools in the study of the cytoskeleton is limited by some of the intrinsic properties of the proteins that comprise it (Table 1), such as their tendency to form highly stable, insoluble structures that aggregate with many proteins, hindering the identification of direct binding partners (Choi et al., 2014).

**TABLE 1:** Routinely used PPI assays and their limitations for studying cytoskeletal proteins.

PPI approach	Description	Limitation
Peptide microarray	An array consisting of thousands of different peptides is incubated with a candidate protein	Uses short linear motifs or protein recognition modules, rather than full-length proteins
Yeast two-hybrid (Y2H)	Physical interaction between two proteins of interest leads to activation of a reporter gene in a yeast cell model	Requires tagging proteins with fragments of a reporter protein, which can introduce steric hindrance and ultimately affect binding. Quantitative methods typically involve additional assays such as flow cytometry
Protein overlay assay (dot blot)	Proteins are spotted on a membrane, incubated with bait, and detected using antibodies against the bait	Requires extremely pure protein samples because there will be no separation by SDS-PAGE. Prey quantification is typically not included in the assay, preventing a comparison of bait affinity between two samples
Affinity purification	Biochemical column-based assay to identify binding partners from lysates or complex mixtures	Cytoskeletal proteins tend to aggregate even during lysis, preventing isolated proteins from interacting in these assays. Quantitative methods typically involve mass spectrometry
Pull-down assay		
Co-IP		
FRET	Proximity ligation for live cell imaging, using a reporter signal	Relying on proximity to infer binding. Not an ideal assay when working with structural proteins, where proximity does not always imply interaction
BRET		
APEX		

Western blots (WBs), first developed in the 1970s, are now among the most widely used assays in biological research for quantifying individual proteins of interest. Far Western blots (FWBs), developed in the 1980s, are an adaptation of WBs for the quantification of PPIs (Wu et al., 2007). FWBs are particularly useful when working with structural proteins, where proximity ligation or other cellular assays can be uninformative, due to individual proteins being tethered to the highly integrated cytoskeletal proteome (Fletcher and Mullins, 2010).

**FIGURE 1:**
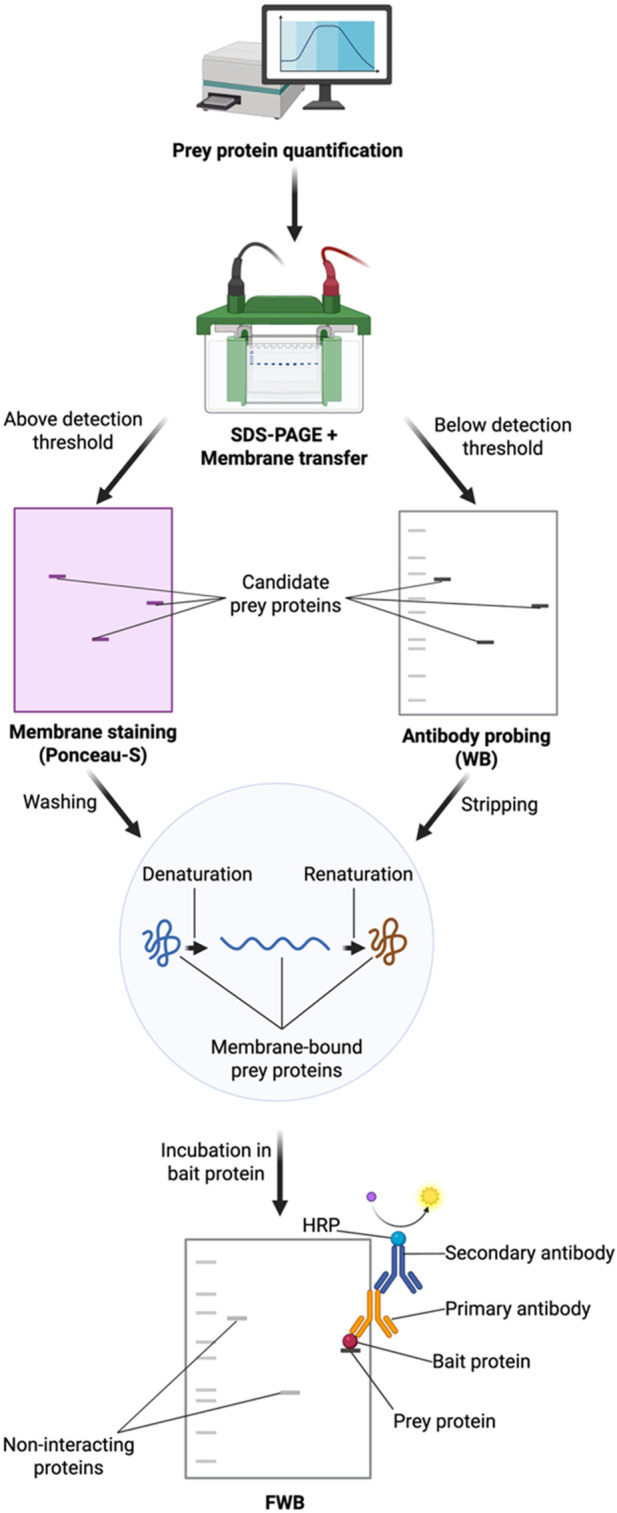
Schematic of the far Western blot (FWB) protocol. Visual overview of the FWB workflow. The assay begins with the quantification of the prey protein (e.g., BCA or NanoOrange™), which is a critical step that determines which loading control strategies are suitable for downstream binding affinity comparisons. Following quantification, samples are loaded onto a gel, separated via SDS-PAGE, and transferred to a membrane, as in a standard Western blot (WB). If the amount of loaded prey protein exceeds the detection threshold of the membrane stain (e.g., Ponceau-S), as determined during the initial quantification, then the membrane is stained, and the membrane-bound proteins are quantified. If the prey protein is below the detection threshold, the membrane is instead blocked and processed through a WB-based approach to quantify membrane-bound proteins. Stained membranes are subsequently washed, and membranes used for WBs are stripped of antibodies. From this point onward, the protocol proceeds identically regardless of the loading control method used. Prior to continuing with the FWBs, the membrane-bound proteins are denatured and renatured. In contrast to WBs, the primary antibody in FWBs does not detect membrane-bound proteins; instead, it detects the bait protein bound to the membrane. A bait protein band appears at the position corresponding to the interacting prey protein, as indicated by the molecular weight marker, while noninteracting prey proteins are not detected during development. (Created with BioRender.com).

Both WBs and FWBs begin with the loading of protein samples (that have been quantified; see “*Protein purification and sample preparation*” below) and running them on an SDS-PAGE gel for size separation. The samples are then transferred to a membrane (e.g., nitrocellulose or polyvinylidene fluoride (PVDF); see “*II. Transfer:* step 8”). In WBs, a primary antibody recognizes the protein of interest on the membrane. In contrast, in FWBs, membrane-bound “prey” proteins denatured during SDS-PAGE separation are detected, and the same blot is then processed to renature the adherent proteins. This renaturation step ensures that the proteins adopt their native conformations, which are essential for bait–prey protein interactions (see “Denaturing/renaturing of prey proteins”). After renaturation, the blot is incubated with the “bait” protein, which is subsequently detected with an antibody against the bait (Figure 1). After application of the secondary antibody, the resultant FWB band reflects the binding of the bait protein to the prey protein on the membrane.

Importantly, the strength of the bait–prey binding affects the intensity of the resultant band. One specific application of FWBs is determining how a mutation in a protein of interest (prey) affects its affinity for a binding partner (bait). In this case, mutant and wild-type proteins are run on separate lanes in the same gel. After transfer, the membrane is incubated with the bait protein, allowing for competitive binding between the mutant and the wild-type prey proteins. The resulting band intensities in the mutant vs. wild-type lanes reflect the relative prey–bait binding affinity. As with other PPI approaches mentioned above, FWBs are limited by the strength of the interaction between the prey and bait proteins, generally requiring moderate to strong interactions. Studying weak or transient protein interactions may require more sensitive approaches, such as confocal scanning (PPI-CONA), a mechanically transduced immunosorbent assay (METRIS), fluorescence polarization (FP), or massively parallel sequencing (MP3-seq) (Du, 2015; Petell et al., 2021; Baryshev et al., 2024; Koszela et al., 2024).

This protocol has been adapted to quantify the relative binding strength of interaction for challenging proteins such as cytoskeletal proteins. The incorporation of a WB on the same membrane later used for a FWB increases the sensitivity of the assay beyond that offered by traditional membrane staining, expanding the application of FWBs to proteins that are not commercially available.

## Materials and equipment

### Reagents

- Purified prey proteins
- Purified bait protein
- Guanidine-HCl (Research Products International, Cat. G49000, Mount Prospect, IL, United States)
- Sodium chloride (Research Products International, Cat. S23020, Mount Prospect, IL, United States)
- Tris (1 M), pH 8.0, RNase-free (Invitrogen, Cat. AM9855G, Carlsbad, CA, United States)
- EDTA (0.5 M), pH 8.0, RNase-free (Invitrogen, Cat. AM9260G, Carlsbad, CA, United States)
- Immobilon®-P Membrane (PVDF) (Millipore Sigma, Cat. IPVH00005, Burlington, MA, United States)
- Primary antibodies:
  - ◦ c-Myc Polyclonal Antibody (1:500) (GenScript, Cat. A00172-40, Piscataway, NJ, United States)
  - ◦ HA Tag Monoclonal Antibody (1:2500) (Invitrogen, Cat. 26183; Carlsbad, CA, United States)
- Secondary antibodies:
  - ◦ Goat-Anti-Rabbit IgG (H + L)-HRP Conjugate (1:2000) (Bio-Rad, Cat. 1706515; Hercules, CA, United States)
  - ◦ Goat-Anti-Mouse IgG (H + L)-HRP-Conjugate (1:2000) (Bio-Rad, Cat. 1706516, Hercules, CA, United States)
- PageRuler™ Plus Prestained Protein Ladder (Thermo Fisher Scientific, Cat. 26619, Waltham, MA, United States)
- Tris Base Ultra Pure (Research Products International, Cat. T60040, Mount Prospect, IL, United States)
- Glycine (Crystalline/Certified) (Thermo Fisher Scientific, Cat. G46-500; Waltham, MA, United States)
- AccuGENE® 10% SDS (Lonza, Cat. 51213; Basel, Switzerland)
- Skim milk powder (local grocery store)
- Tween-20 (Research Products International, Cat. P20370, Mount Prospect, IL, United States)
- Pierce™ DTT (dithiothreitol) (Thermo Fisher Scientific, Cat. 20290, Waltham, MA, United States)
- Glycerol (Sigma-Aldrich, Cat. G7893, Saint Louis, MO, United States)
- Bond-Breaker™ TCEP Solution (Thermo Fisher Scientific, Cat. 77720; Waltham, MA, United States)
- Methanol (Certified ACS) (Thermo Fisher Scientific, Cat. A412-4, Waltham, MA, United States)
- NuPAGE™ LDS Sample Buffer (4×) (Invitrogen, Cat. NP00007, Carlsbad, CA, United States)
- NuPAGE™ MOPS SDS Running Buffer (20×) (Invitrogen, Cat. NP0001, Carlsbad, CA, United States)
- SuperSignal™ West Pico PLUS Chemiluminescent Substrate (Thermo Fisher Scientific, Cat. 34580, Waltham, MA, United States)
- Restore™ Western blot Stripping Buffer (Thermo Fisher Scientific, Cat. 21059, Waltham, MA, United States)
- Bis-Tris Mini Protein Gel, 4%–12%, 1.0–1.5 mm (Invitrogen, NP0335BOX, Carlsbad, CA, United States)
- Tris-buffered saline (TBS) (10×) (Research Products International, T60075, Mount Prospect, IL, United States)

### Equipment

- iBright™ CL1500 Imaging System (Invitrogen, Cat. A44116, Carlsbad, CA, United States)
- Mini Gel Tank (Invitrogen, Cat. A25977, Carlsbad, CA, United States)
- Trans-Blot® SD Semi-Dry Electrophoretic Transfer Cell (Bio-Rad, Cat. 1703940, Hercules, CA, United States)
- ImageJ (National Institutes of Health, Bethesda, MD, United States) (Schneider et al., 2012)

## Methods

### Experimental design

#### Protein purification and sample preparation

When studying cytokeratin proteins, using purified samples rather than lysates is critical to ensure that the proteins are not already bound prior to initiating the assay. Protein purification is not described here, but has been extensively described in the literature (Safarik and Safarikova, 2004). An important consideration to implement in an experimental design is to include tags in the plasmids if specific antibodies are not available. Opting for epitope tags, such as His, c-Myc, or HA, is beneficial because of their small size (minimizing steric hindrance) and the availability of specific antibodies and antibody-coated magnetic beads. Fluorescent tags pose a higher risk of disrupting PPIs due to steric hindrance or aggregation (especially with GFP), thereby diminishing protein interactions (Thorn, 2017).

**FIGURE 2:**
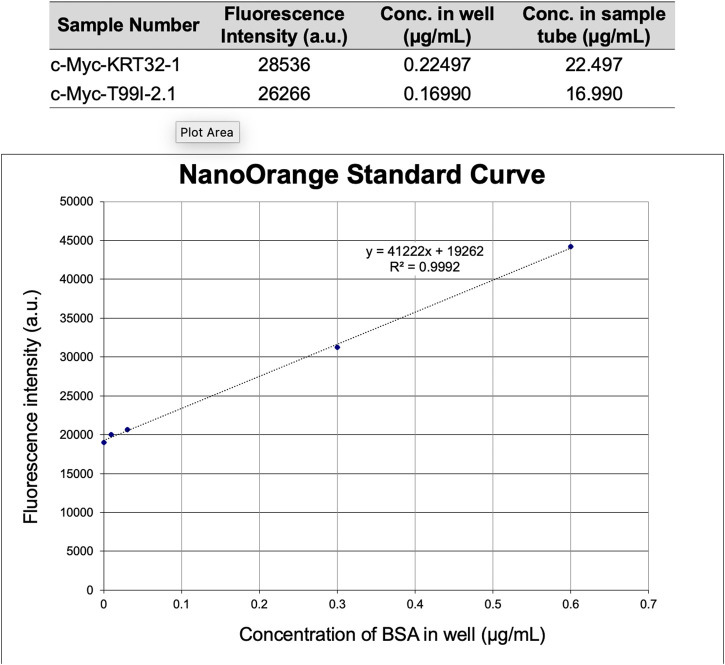
Prey protein sample quantification. Prey proteins were quantified using NanoOrange™ (Thermo Fisher Scientific) on a Tecan Infinite M200 PRO (Tecan) fluorescent plate reader. Fluorescent values for each field (prey protein and BSA dilutions) are the average of three technical replicates.

A critical step is to have a sufficient amount of the purified protein. This FWB protocol has been successful using as little as 100 ng of prey protein and 200 ng of the bait protein in purified forms. It is important to quantify the samples prior to initiating the assay to guarantee equal loading of all wells and to determine the normalization method. Several methods for protein quantification exist. A colorimetric spectrometer-based BCA assay and fluorometric NanoOrange™ (for more dilute samples) are both well-validated options (Figure 2). If the assay is performed with less than 200 ng of prey proteins, which is below the threshold for using Ponceau-S for quantification of sample loading, it becomes necessary to probe the prey proteins after the membrane transfer and before incubation in the bait (i.e., a traditional WB must be conducted for the prey protein first). Primaries for the bait (in the WB) and bait (in the FWB) must have different hosts to prevent interactions between the secondary antibody for the bait and the primary antibody for the prey. Reversible stain options are discussed in Table 2 and compared to WBs. When possible, increasing the amount of protein loaded is recommended, as it eliminates the requirement for a WB.

**TABLE 2:** Sensitivity of different membrane-bound protein detection methods.

Membrane-bound protein detection method	Sensitivity range
Ponceau-S	>200 ng
BLOT-*FastStain* ^TM^	>0.5 ng (BSA)
Swift™ membrane stain	>0.5 ng (BSA)
WB	(antibody-dependent)

#### Standard controls

A positive prey control (i.e., a known interacting protein) should be included. A negative control (i.e., known noninteracting proteins), such as BSA, can also be incorporated in the form of prey proteins.

#### Denaturing/renaturing of prey proteins

SDS-PAGE denatures proteins during separation. During transfer, proteins typically renature as SDS is eliminated. Since FWB is a PPI assay, it is crucial that the prey proteins have the correct 3D conformation before the bait is introduced. To ensure that the transferred proteins (and most importantly, their binding sites) are folded correctly during the transfer, it is recommended to do a denaturing step in guanidine hydrochloride, followed by a renaturing step, which is accomplished by gradually decreasing the concentration of guanidine hydrochloride. It is important to note that although uncommon in small proteins (<50 amino acids), protein misfolding and aggregation have been reported in some large proteins (>200 amino acids). Protein misfolding can eliminate native binding domains or introduce novel ones (Devi et al., 2022; Mecha et al., 2022). This event is partially due to environmental factors (e.g., pH, temperature, salt, and pressure), and aggregation and folding processes occurring in parallel, where more complex proteins (slow folding) are unable to overcome the aggregation rate, unless folding occurs independently and simultaneously in different domains. Some proteins also require specific chaperones and co-chaperones, absent in purified protein samples, to facilitate proper folding by binding and stabilizing folding intermediates (Castelli et al., 2024). The binding of ligands to folding intermediates is also known to affect the rate and efficiency of folding by inducing conformational changes and guiding the protein to a more favorable folding route. Researchers have exploited this phenomenon and developed pharmacological chaperones (PCs) that have improved folding efficiency in both *in vitro* and *in vivo* models (Leidenheimer and Ryder, 2014; Kasper and Park, 2015). To determine if prey proteins are subject to misfolding during renaturation, it is recommended to perform preliminary blots using known interacting proteins (positive controls) and noninteracting proteins (negative controls) as bait, testing for the presence of nonspecific binding to negative controls or the absence of binding to positive controls. Prey proteins that demonstrate misfolding during renaturation are not suitable for FWBs.

#### Detection

Although fluorophore-conjugated secondary antibodies allow for the simultaneous detection of multiple prey proteins, simplifying the assay, chemiluminescence-based detection is favored due to its higher sensitivity (Gingrich et al., 2000; Ha et al., 2025). This involves incubating the membrane with a substrate that emits light upon reaction with horseradish peroxidase (HRP), which is conjugated to the secondary antibody. The emitted light is then detected in this protocol by iBright™. Band intensities are quantified with FIJI/ImageJ (Table 5).

We prefer HRP-conjugated secondary antibodies because they are more easily stripped for subsequent reprobing with additional antibodies, in addition to having a higher threshold of detection than fluorescent labels.

The sensitivity range for FWBs is context-dependent and influenced by numerous factors, including protein abundance, membrane selection, and antibody affinity. Titration of these parameters can be implemented as a preliminary step to determine the sensitivity range for the selected combination of proteins and reagents.

### Procedure

#### Sample preparation and gel loading/running

1. Loading buffer should be made fresh before each use. The amount required for the entire gel has to be calculated, and a stock solution prepared for the entire assay.
• Suggested loading buffer composition:
  ◦ 1 part Bond-Breaker TCEP Solution
  ◦ 9 parts NuPAGE LDS Sample Buffer (4×)
2. The prey protein must be diluted in an equal volume of loading buffer before loading the gel, keeping in mind the maximum loading volume per well.
3. Prey protein concentration must be adjusted so that a suitable amount of protein is loaded onto the gel (dependent on antibody affinity). An equal volume of prey proteins should be loaded in each well so that samples run uniformly.
4. An equal volume of prey protein and loading buffer must be mixed in a 1.5 mL microcentrifuge tube, incubating them for 5 min at 95 °C on a heat block.
5. A molecular weight ladder needs to be loaded.
6. The gel must be run in 1× MOPS buffer at 80 V for 20 min, then increasing the voltage to 125 V for a period of time depending on the position of the prey protein on the gel, which is monitored by a prestained protein ladder. The ideal pattern is for the prey proteins to remain in the middle of the gel.

*Note: The position of the corresponding marker in the ladder needs to be monitored to prevent the prey proteins from running off the gel.

#### Transfer

7. The gels must be left in the buffer until ready to transfer.
8. The PVDF membrane is to be activated by incubating it in a dish with methanol for 5 s.

*Note: Membranes must always be handled carefully with tweezers, avoiding contact with any surfaces.

**Note: Although nitrocellulose membranes can also be used, we generally recommend PVDF because: (a) binding of proteins to the membrane occurs mainly through hydrophobic interactions (Gibbons, 2014). Because PVDF is more hydrophobic, PVDF membranes have a higher binding capacity and bind proteins more tightly, making them more suitable for working with less concentrated samples (Gibbons, 2014), (b) PVDF membranes are also more chemically resistant than nitrocellulose, allowing for antibody stripping and repeated probing (Xiang et al., 2021), and (c) PVDF membranes are well-suited to high molecular weight proteins, such as cytoskeletal proteins (Xiang et al., 2021).

9. The activated PVDF membrane and transfer pads must be transferred to a dish containing 1× transfer buffer.
• Suggested transfer buffer recipe:
  ◦ 10× transfer buffer stock: 58.2 g Tris base +144.1 g glycine +39.5 mL 10% SDS solution + fill to 1 L with ddH_2_O.
  ◦ 1× transfer buffer: 100 mL 10× stock +200 mL methanol +700 mL ddH_2_O.
10. A transfer sandwich needs to be created using the transfer pads, PVDF membrane, and gel.
11. The transfer needs to run at 13 V for 2 h.

#### Western blot analysis

*This critical step sets the baseline for the downstream analysis of bait protein binding. If running different sets of prey proteins (i.e., wild-type (WT) and mutant) to determine any changes in binding affinity, it is very important that the loading of the different samples is consistent.

**If the prey protein load is over the minimum detection threshold for the membrane stain (i.e., Ponceau-S and Coomassie blue), the blocking and antibody incubations must be skipped and the staining performed once the transfer is complete. Once staining is complete and washed off, the “Denaturing/renaturing of proteins on the membrane” section of the protocol must be followed. Blocking can be skipped at this step when using a membrane stain instead of WB probing, as the membranes will be re-blocked prior to anti-bait antibody incubation.

***WBs are required if the prey protein load is under the detection threshold for membrane stains. Since the same membrane will be used later for FWBs, the primary antibodies selected for the prey and bait must have different hosts.

**FIGURE 3:**
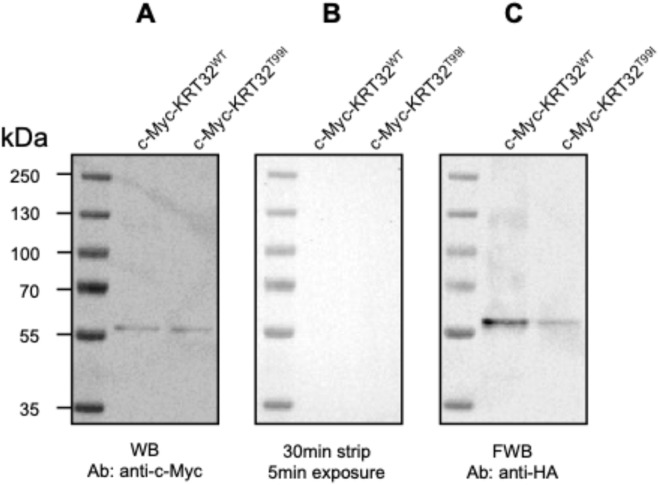
Chemiluminescent blot development. Blots from one experiment (single membrane) using c-Myc-KRT32^WT^ and c-Myc-KRT32^T99I^ as prey. **(A)** Western blot analysis using primary antibodies against the c-Myc tag. Bands for both prey proteins were analyzed using ImageJ to confirm even loading (see *III. Western blot analysis*, step 18). **(B)** A 5-min exposure following 30 min of antibody stripping at RT using stripping buffer, showing complete removal of secondary antibodies (see *IV. Membrane stripping*, step 23). **(C)** Far Western blot analysis using anti-HA antibodies, post-denature/renature, and incubation with bait protein (HA-KRT82) (see *VIII. Detection of bait proteins bound to prey proteins on the blot*, step 37). Band quantification using ImageJ reveals a stronger signal from the WT prey, indicating a reduced binding affinity in the T99I construct. All blots were developed using iBright™ chemiluminescence.

12. The transferred membrane must be blocked using 5% non-fat milk in 1× TBS-T for 1 h at room temperature (RT) with agitation.
13. The primary antibody must be incubated against the prey protein (diluted in 5% milk in TBS-T) overnight at 4 °C with agitation (pause point), following the manufacturer’s recommendations for dilution.
• If needed, this step can be shortened to 4 h at RT.
14. The primary antibody must be washed off with TBS-T for 5 min at RT with agitation (to be repeated three times).
15. Incubating in an HRP-conjugated secondary antibody (diluted in 5% milk in TBS-T) for 4 h at RT with agitation, following the manufacturer’s recommendations for dilution.
16. The secondary antibody must be washed off with TBS-T for 5 min at RT with agitation (to be repeated three times).
17. The membrane is to be incubated in the chemiluminescent substrate for 10 min at RT with agitation, protected from light.
18. Chemiluminescence development must be performed on the iBright™ immediately (Figure 3A).
• It must be verified that none of the bands on the gel are saturated. If saturation is detected, the exposure time must be reduced and reimaged. Images must be saved as RAW files.
19. Quantifying all prey bands on ImageJ using the “analyze > gel > select lane” function.

#### Membrane stripping

20. Once the prey proteins have been imaged, the secondary antibodies must be stripped off the membrane for 30 min at RT with agitation using the stripping buffer.
21. The stripping buffer must be washed off with TBS-T for 5 min at RT with agitation (to be repeated three times).
22. The membrane is to be re-incubated in chemiluminescent substrate for 10 min at RT with agitation, protected from light.
23. Complete secondary antibody stripping must be verified by performing a 5 min exposure to iBright™ (Figure 3B).
24. The chemiluminescent substrate must be washed off with TBS-T for 5 min at RT with agitation (to be repeated three times).

#### Denaturing/renaturing of proteins on the membrane

25. Proteins must be denatured and renatured on the membrane in AC buffer by gradually reducing the guanidine-HCl concentration.
26. Guanidine-HCl buffers must be made fresh every time and should be made while the transfer is ongoing to avoid having the membrane dry out after the transfer is complete.
27. Buffer recipes for 25 mL working volumes can be found in Table 3. All five buffer solutions must be stored at 4 °C until needed. The highlighted reagents are the only ones that vary in volume among all five solutions.
• All incubations must be performed on a rocker.
• Membrane incubation in AC buffer containing no guanidine-HC is to be carried out overnight at 4 °C (pause point).
  ◦ If needed, this step can be shortened to 1 h at 4 °C.

**TABLE 3:** Recipes for denaturing/renaturing buffers.

Concentration of guanidine-HCl (M)	6	3	1	0.1	0
Glycerol (mL) (final 10%)	2.5	2.5	2.5	2.5	2.5
5 M NaCl (mL) (final 100 mM)	0.5	0.5	0.5	0.5	0.5
1 M Tris, pH 8 (mL) (final 20 mM)	0.5	0.5	0.5	0.5	0.5
0.5 M EDTA (mL) (final 1 mM)	0.05	0.05	0.05	0.05	0.05
10% Tween-20 (mL) (final 0.1%)	0.25	0.25	0.25	0.25	0.25
Guanidine-HCl (8 M) (mL)	18.75	9.30	3.13	0.31	0
Milk powder (g) (final 2%)	0.5	0.5	0.5	0.5	0.5
1 M DTT (μL) (final 1 mM)	25	25	25	25	25
ddH_2_O (mL)	2.45	12.82	18.07	20.89	21.20
Total volume (mL)	25	25	25	25	25
Time/temperature	30 min/room temperature (RT)	30 min/RT	30 min/RT	30 min/4 °C	1 h overnight/4 °C

#### Blocking

28. Once the denature and renature incubations are complete, the membrane must be blocked by incubating it with 5% milk in TBS-T blocking solution for 1 h at RT with agitation.

#### Incubation of the membrane with the purified interacting (bait) protein(s)

29. While the membrane is blocking, the protein-binding buffer has to be prepared (composition is in Table 4).
30. The membrane needs to be incubated with the purified bait protein (minimum of 200 ng) in 5 mL of the reaction buffer for 3 h at RT with agitation (alternatively, overnight at 4 °C).

**TABLE 4:** Protein-binding buffer composition.

Protein-binding buffer composition
100 mM NaCl
20 mM Tris, pH 8
0.5 mM EDTA
10% glycerol
0.1% Tween-20
2% milk powder
1 mM DTT

#### Detection of bait proteins bound to prey proteins on the blot

31. The unbound bait protein needs to be washed off with TBS-T for 5 min at RT, with agitation, to be repeated three times (a total of four washes).
32. The blot with the primary antibody, which is diluted in 5% milk in TBS-T, must be incubated for 4 h at RT with agitation.
  - Can be carried out overnight at 4 °C if needed.
33. A wash with the TBS-T must be performed for 5 min at RT, with agitation, to be repeated three times (a total of four washes).
34. Incubation must follow with an HRP-conjugated secondary antibody diluted in 5% milk in TBS-T for 2 h at RT, with agitation.
35. A wash must be performed with TBS-T for 5 min at RT with agitation, to be repeated three times (a total of four washes).
36. The membrane in the chemiluminescent substrate must be incubated for 10 min at RT with agitation, protected from light.
37. The blot needs to be developed immediately on the iBright™ to visualize the position of the bait protein (Figure 3C).
38. The bait band intensities must be quantified on ImageJ.

*Note: As previously mentioned, we do not recommend the use of cell lysates in FWBs, but if cell lysates are used as prey instead of purified protein, it is crucial to confirm the identity of the prey proteins interacting with the bait. Relative size compared to the molecular weight ladder is not sufficient because it does not exclude other proteins of a similar size that are present in the lysate. Edman degradation and mass spectrometry (MS) are examples of assays that can be implemented for prey protein identification.

## Results

To illustrate the application of far Western blotting, we present results from a binding affinity comparison between the wild-type (WT) keratin 32 (KRT32^WT^) and a mutant protein containing a missense variant p.Thr99Ile (KRT32^T99I^), to keratin 82 (KRT82). The variant p.Thr99Ile was identified as a likely pathogenic heterozygous variant in a large kindred with loose anagen hair syndrome (LAHS) in successive generations (autosomal dominant), and it segregated appropriately with the phenotype. Keratin 32 is a type I hair keratin expressed in the cuticle layer of the hair shaft, known to heterodimerize with type II hair keratin 82 (Melo et al., 2025).

Keratins are cytoskeletal proteins, predominantly expressed in epithelial cells, that are essential for mechanical stability, cellular integrity, and intracellular signaling. Pathogenic variants in keratin genes have been implicated in several human diseases, including skin and liver disorders (Toivola et al., 2015). There are 54 human keratins, which are further divided into type I and type II (Moll et al., 2008). High sequence homology between keratins prevents the development of antibodies specific enough to discriminate between different keratins. Type I and type II keratins form heterodimers that are precursors to intermediate filaments (IFs) (Jacob et al., 2018). Keratins are extremely promiscuous, with type I proteins able to bind to any type II and *vice versa* (Hatzfeld and Franke, 1985). These properties, along with the high frequency of disulfide bonds, often result in type I and type II keratins co-purifying from cell lysates. Thus far, *in vitro* PPI studies have been limited by the unavailability of specific antibodies for many keratins and the difficulty of purifying these proteins (Table 1).

For this study, KRT32^WT^ and KRT32^T99I^ were used as prey proteins, while KRT82 was used as bait. HEK293 cells were transfected with the c-Myc-KRT32^WT^, c-Myc-KRT32^T99I^, or HA-KRT82^WT^ plasmid. The tagged proteins were purified from the lysates using anti-c-Myc magnetic beads (Pierce™ anti-c-Myc magnetic beads, Thermo Fisher Scientific) or anti-HA magnetic beads (Pierce™ anti-HA magnetic beads, Thermo Fisher Scientific) for KRT32 and KRT82, respectively. Purified samples underwent Western blotting to verify the presence of the protein of interest in each sample, as confirmed by molecular weight. Incorporating the c-Myc and HA tags in the plasmid design allowed for the use of precoated magnetic beads in the purification step and highly specific anti-c-Myc and anti-HA primary antibodies.

Developed membrane images showed similar loading of the c-Myc-KRT32 prey proteins (∼60 kDa), as revealed with an anti-c-Myc WB (Figure 3A). This was followed by complete stripping of the secondary antibody for anti-c-Myc (Figure 3B). Next, the membrane was incubated with purified HA-KRT82, and an anti-HA primary antibody was applied. After applying a secondary antibody for anti-HA, the resulting FWB membrane showed bands at ∼60 kDa that reflect binding between KRT32 and KRT82. This band is substantially weaker for KRT32^T99I^ than for KRT32^WT^ (Figure 3C), revealing that the threonine-to-isoleucine substitution at amino acid 99 in keratin 32 lowers its binding affinity for keratin 82. Four biological replicates of this assay were performed, showing a 20.7% reduction in band intensity for the c-Myc-KRT32^T99I^ compared to the c-Myc-KRT32^WT^ band (Melo et al., 2025). Bands were quantified using the “analyze > gels > plot lanes” function in ImageJ, which allows for band selection and signal quantification. Bands from bait probing (Figure 3C) were normalized to their respective bands from prey probing (Figure 3A), and the KRT32^WT^ and KRT32^T99I^ lanes were compared (Table 5).

**TABLE 5:** Quantification of band intensities (ImageJ).

Band	c-Myc-KRT32^WT^	c-Myc-KRT32^T99I^
WB	1442.971	826.435
FWB	3622.770	988.870
FWB/WB	2.511	1.197

Several hurdles were encountered during the adaptation of this protocol. Most issues occurred in the initial steps of the assay (protein purification through membrane stripping). Table 6 includes some of the most common issues, potential causes, and solutions.

**TABLE 6:** Troubleshooting.

Step	Issue	Potential reason	Troubleshooting steps
18–19	Absent prey protein bands on WB	Prey protein may be absent from the solution, the sample might be too dilute, or the primary antibody binds too weakly to the prey	• Increase protein load • Repurify samples • Increase antibody concentration • Include an epitope tag (e.g., c-Myc or HA) in the protein construct • Change the primary antibody
18–19	Prey protein antibody detects band(s) heavier than the reported size in the literature	The prey protein in stock is likely aggregating, interacting with a protein in solution, homodimerizing, or undergoing extensive post-translational modifications. A different isoform of the prey protein could be present in the stock	• If the band at the correct size is also present, cut off the upper portion of the membrane. This prevents the already-bound prey proteins from interfering with quantification • If the band at the correct size is too faint or absent, add TCEP to the loading buffer containing the prey protein, and boil the samples prior to SDS-PAGE separation • Include lysates of a different cell line
18–19	Prey protein antibody detecting band(s) below the reported size in the literature	Prey protein in stock is degraded. A different isoform of the prey protein could be present in the stock	• If the band at the correct size is also present, cut off the lower portion of the membrane. This removes degraded proteins from the bait-binding portion of the assay • If the band at the correct size is too faint or absent, repurify the prey protein • Include a protease inhibitor cocktail in the lysis buffer • Include lysates of a different cell line of primary cells
18–19	Prey protein bands are too thick or lack defined boundaries, hindering accurate quantification	The prey protein stock may be insufficiently pure, or contaminants may be present in the solution	• Add TCEP to the loading buffer containing the prey protein and boil the samples prior to SDS-PAGE separation • If the issue persists, repurification may be required
18–19	Prey proteins WB membrane development shows V-shaped bands	Uneven separation during SDS-PAGE or uneven loading volume across wells	• Confirm that equal volumes were loaded across all wells • Run the gel at a lower voltage for a longer duration • If the issue persists, refrigerate the running buffer prior to use and consider running the SDS-separation at 4 °C
23	Post-strip prey protein bands are still visible after 5 min exposure	Insufficient stripping of the anti-prey secondary antibody from the membrane	• Increase the incubation time in the stripping buffer • Perform stripping on a rocker at 37 °C • Use a different stripping buffer

## Discussion

While various techniques have advanced PPI research, cytoskeletal proteins pose unique challenges to the use of many PPI research methods due to their tendency to form large, stable, and insoluble aggregates. This study highlights the potential of FWB as an effective tool for studying PPIs involving cytoskeletal proteins. FWB is particularly useful for studying structural proteins that are difficult to analyze using other methods, offering a streamlined approach by detecting binding partners based on molecular weight. This is especially valuable when the cellular context or fixed intracellular location limits other techniques.

Additionally, FWB is useful for studying the effect of gene variants on protein interactions, providing insights into diseases caused by disruptions in cytoskeletal protein interactions. By comparing wild-type and variant proteins, as illustrated with the keratin case study, FWB helps researchers understand how subtle changes in protein structure can contribute to disease phenotypes.

In conclusion, FWB provides a powerful and broadly applicable approach for PPI analysis in laboratories with standard biochemical capabilities. Beyond its utility for cytoskeletal proteins, it is broadly applicable to structural or insoluble proteins and to proteins lacking specific antibodies. As such, FWB represents a valuable addition to the biochemical research toolbox for any laboratory studying protein biology.

## Data Availability

The raw data supporting the conclusions of this article will be made available by the authors, without undue reservation.
